# Application of a semi-automated dicentric scoring system in triage and monitoring occupational radiation exposure

**DOI:** 10.3389/fpubh.2022.1002501

**Published:** 2022-10-20

**Authors:** Younghyun Lee, Seung Hyun Kim, Yang Hee Lee, Su San Yang, Hyo Jin Yoon, Ruth C. Wilkins, Seongjae Jang

**Affiliations:** ^1^Laboratory of Biological Dosimetry, National Radiation Emergency Medical Center, Korea Institute of Radiological and Medical Sciences, Seoul, South Korea; ^2^Department of Biomedical Laboratory Science, College of Medical Sciences, Soonchunhyang University, Asan, South Korea; ^3^Consumer and Clinical Radiation Protection Bureau, Health Canada, Ottawa, ON, Canada

**Keywords:** dicentric chromosome assay, radiation biodosimetry, semi-automated scoring, radiological emergencies, occupational radiation exposure

## Abstract

The dicentric chromosome assay (DCA) is considered the gold standard for radiation biodosimetry, but it is limited by its long dicentric scoring time and need for skilled scorers. The automation of scoring dicentrics has been considered a strategy to overcome the constraints of DCA. However, the studies on automated scoring methods are limited compared to those on conventional manual DCA. Our study aims to assess the performance of a semi-automated scoring method for DCA using *ex vivo* and *in vivo* irradiated samples. Dose estimations of 39 blind samples irradiated *ex vivo* and 35 industrial radiographers occupationally exposed *in vivo* were estimated using the manual and semi-automated scoring methods and subsequently compared. The semi-automated scoring method, which removed the false positives of automated scoring using the dicentric chromosome (DC) scoring algorithm, had an accuracy of 94.9% in the *ex vivo* irradiated samples. It also had more than 90% accuracy, sensitivity, and specificity to distinguish binary dose categories reflecting clinical, diagnostic, and epidemiological significance. These data were comparable to those of manual DCA. Moreover, Cohen's kappa statistic and McNemar's test showed a substantial agreement between the two methods for categorizing *in vivo* samples into never and ever radiation exposure. There was also a significant correlation between the two methods. Despite of comparable results with two methods, lower sensitivity of semi-automated scoring method could be limited to assess various radiation exposures. Taken together, our findings show the semi-automated scoring method can provide accurate dose estimation rapidly, and can be useful as an alternative to manual DCA for biodosimetry in large-scale accidents or cases to monitor radiation exposure of radiation workers.

## Introduction

Human exposure to ionizing radiation (IR) is inevitable, as IR is widely used in medical, industrial, military, and research applications. Radiation-induced cytogenetic aberrations have been used for dose assessments (1). Of these assessments, the dicentric chromosome assay (DCA) is considered the gold standard for radiation biodosimetry due to its radiation-specificity and low background level of dicentrics (2–4). Dicentric chromosome assay has been used to estimate radiation doses in humans exposed to radiological accidents, including Chernobyl, Goiania, and Tokaimura (5–8). However, conventional manual-scoring DCA is unsuitable for large-scale radiological accidents because of its time-consuming and laborious process of scoring and sample processing, given that handling a large number of individual blood samples would be necessary in such cases (9).

Various approaches have been performed to overcome the constraints of DCA. For rapid dicentric scoring, Flegal et al. (10) introduced DCA QuickScan as an alternative scoring method for triage. Automated scoring systems, such as the dicentric chromosome scoring algorithm (DCScore) (Metafer, USA) and the Automated Dicentric Chromosome Identifier and Dose Estimator (ADCI) (CytoGnomix, Canada) (11, 12), as well as an Automated platforms for cell harvesting and chromosome preparation such as HANABI-PI Metaphase Chromosome Harvester (ADS Biotec, USA) and the Rapid Automated Biodosimetry Tool (RABiT) (Columbia University, USA), have also been developed to minimize human intervention (13, 14). In addition, Balajee et al. (15) developed a miniaturized version of DCA for radiological triage by using barcoded 1.4 ml tubes and DCScore to reduce sample processing time and scoring time. Moquet et al. (16) also suggested lyse/fix method for high-throughput sample processing in radiation biodosimetry.

The automation of dicentric scoring has reduced scoring time and enhanced DCA capacity (9). DCScore is a representative automated scoring tool for rapid DCA. Dicentric scoring with DCScore is based on the Metafer platform, which is widely used in many cytogenetic laboratories and hospitals for finding metaphases. DCScore detects dicentrics in well-spread metaphases matching classification criteria (i.e., width, area, and number of chromosomes), but it is somewhat different from manual DCA in terms of scoring criteria (9, 11). For example, metaphases including more than 45 chromosomes are analyzed in manual DCA, the wider range (i.e., more than 40, which can be different) could be acceptable in DCScore-based scoring. Given that there are differences between the two methods, the performance of DCScore should be validated for its application. The performance of manual DCA has been well-studied for radiation biodosimetry (17–20), but relatively few studies have validated DCScore. In most of these studies, the performance of DCScore has been evaluated in triage using *ex vivo* irradiated blood (9, 21, 22); however, the application of DCScore in estimating the accurate radiation dose in *in vivo* irradiated humans has not been reported.

Our group has performed DCA to estimate the radiation dose in potentially exposed individuals, including radiation workers. Currently, we established a semi-automated scoring method with DCScore software for rapid dose assessment. In the present study, the performance of the semi-automated scoring method was investigated in *ex vivo* irradiated blind samples and *in vivo* occupationally exposed blood lymphocytes to explore the application of this automated scoring approach in triage and monitoring occupational radiation exposure.

## Materials and methods

### Subjects and sample preparation

Blood samples were collected with the informed consent of the donors, and all experiments were performed in accordance with the ethical standards of the Institutional Review Board (IRB no. K-1301-002-033, KIRAMS 2018-03-005, REB 2002-0012). To construct the dose-response curves, blood samples were collected from two healthy donors and irradiated with different doses of Co-60 (0, 0.1, 0.25, 0.5, 0.75, 1, 2, 3, and 4 Gy) using the GammaBeam 100-80 (Best Theratronics, Canada) of the Korea Institute of Radiological and Medical Sciences (KIRAMS) at a dose rate of 0.5 Gy/min. To compare the performances of the manual and semi-automated scoring methods, images previously analyzed by manual scoring were used (23–25). Blind samples (*n* = 39) irradiated *ex vivo* were prepared in Health Canada for the intercomparison exercises. To validate DCA using *in vivo* irradiated samples, blood samples were collected from 35 industrial radiographers performing non-destructive testing. In order to assess the ability to estimate dose in wide range of radiation dose, we randomly selected workers with relatively high (≥0.1 Gy, *n* = 16) and low (<0.1 Gy, *n* = 19) dose estimates using manual scoring DCA, and their metaphase images were reanalyzed using semi-automated scoring method.

### DCA

Samples were cultured according to the methods recommended by the IAEA (1). Briefly, whole blood was cultured in RPMI 1640 medium (Gibco, Thermo Fisher Scientific, Waltham, MA, USA) supplemented with 20% fetal bovine serum (Gibco), 1% antibiotic-antimycotic (Gibco), and 2% phytohemagglutinin (Gibco) at 37°C for 48 h in a humidified atmosphere of 5% CO_2_ in air. Colcemid solution (Gibco), at a concentration of 0.07 μg/ml, was added to the medium, 24 h before harvesting. Blood samples were treated with 0.075 M KCl for 25 min at 37°C, followed by fixation with cold methanol and acetic acid (3:1). Fixed cells were dropped onto slides, which were stored for 12 h at 60°C. The slides were stained with Giemsa solution (Sigma-Aldrich, St Louis, MO, USA). Metaphase images were captured using Metafer 4 (MetaSystems GmbH, Altlussheim, Germany).

### Analysis of dicentrics

For manual DCA, metaphase images were analyzed by well-trained scorers. A total of 1,000 metaphases or 100 dicentrics were scored in metaphase cells containing more than 45 centromeres. Automatic detection of dicentric candidates was performed using the DCScore software in Metafer. For the semi-automated scoring method, the dicentrics were inspected by well-trained scorers to remove false-positive dicentrics (9).

### Assessment of DCA performance

Dose estimates and their confidence intervals (CIs) were calculated based on the dose-response curve of each scoring method according to IAEA. The calibration curve of manual DCA is as follows: *Y* = 0.00105 (± 0.00010) + 0.0355 (± 0.0041) × *D* + 0.0644 (± 0.0027) × *D*^2^, where *Y* is the yield of dicentrics and *D* is the dose. The curve of semi-automated DCA is described in the Results section with raw data. The accuracy of the reported dose estimates was measured by comparing the delivered and reported doses. When the delivered dose falls within the 95% CI of the dose estimate, or the reported dose is within 0.5 Gy of the delivered dose, dose estimates are considered correct. To evaluate the discriminatory power of DCA, the accuracy, sensitivity, and specificity to distinguish binary categories using the corresponding threshold doses of 0.1 and 1.5 Gy were calculated (26) as follows: Accuracy = (True positive + True negative) × 100/Total; Sensitivity = True positive × 100/(True positive + False negative); Specificity = True negative × 100/(True negative + False positive). The binary categories reflect clinical, diagnostic, and epidemiological significance as follows: Never vs. ever (0 Gy/≥0.1 Gy), to avoid clinical resources being occupied by the “worried well” group; ≤0.1 vs. >0.1 Gy, to distinguish groups such as those who do not need clinical support from others, where deterministic or stochastic effects in adults may occur or become detectable using epidemiological methods; ≤1.5 vs. >1.5 Gy, to identify the group of patients who will likely experience acute radiation syndrome several days after radiation exposure.

### Statistical analysis

Statistical analyses were performed using SPSS ver. 23 (IBM, Armonk, NY, USA) and GraphPad Prism ver. 7 software (GraphPad Software, San Diego, CA, USA). To evaluate the agreement between the manual and semi-automated scoring methods, Cohen's kappa statistics and McNemar's test were performed. Correlations between the two scoring methods were analyzed by Spearman rank correlation. Statistical significance was set at *p* < 0.05.

## Results

### Dose response curve generated by the semi-automatic scoring method

**Figure 1:**
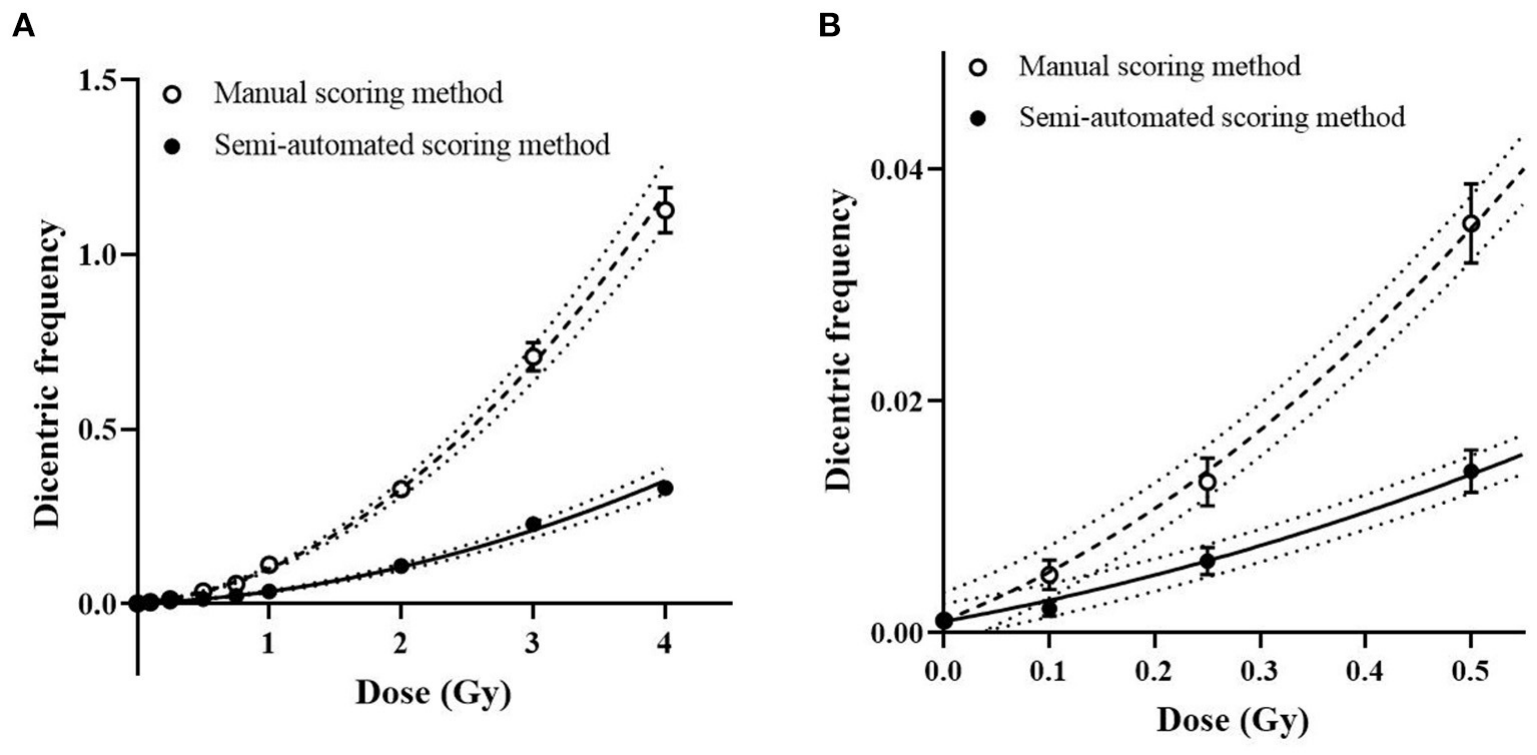
Dose response curve for dicentric chromosome assay (DCA). Symbols and bars represent averaged dicentric yields and standard error by manual (open circles) and semi-automated scoring (filled circles) methods. **(A)** Dose response curves by manual (dashed curve) and semi-automated scoring (straight curve) methods and their 95% confidence intervals (dotted curves) are represented. **(B)** A magnified curve of low-dose region (<0.5 Gy) is shown.

To establish a dose-response curve with the automatic scoring system, the slides produced for the calibration curve of manual DCA were re-analyzed with the DCScore software in Metafer. As a high false positive rate in the automatic scoring system was previously known (9), detected dicentric chromosomes (DC) were confirmed by well-trained scorers. After visual validation, an average of 81 false positives was found in 1,000 cells. Table 1 shows the frequency and distribution of DC in human peripheral lymphocytes for doses from 0 to 4 Gy. The U-values of all samples were lower than 1.96, which indicates that all the dicentric distributions obtained in this semi-automated scoring method followed a Poisson distribution. As shown in Figure 1, the dose response curve was fitted to a linear quadratic model as follows: *Y* = 0.00097 (± 0.00021) + 0.016 (± 0.0022) × *D* + 0.018 (± 0.0012) × *D*^2^, where *Y* is the yield of DC and *D* is the dose. All curve coefficients were statistically significant (*Z*-test, *p* < 0.05), and the χ^2^ goodness-of-fit test was non-significant (*p* = 0.89). The results imply that the linear quadratic model is properly fitted here. The dose response curves for manual and semi-automated scoring methods are shown in Figure 1. The yields of DC by the semi-automated scoring method were lower than those in the manual assay, but a significant correlation between the two methods was observed (correlation coefficient = 0.78, *p* < 0.001).

**Table 1:** Number and distribution of dicentrics in human lymphocytes from blood samples irradiated with Co-60 γ-ray.

**Dose (Gy)**	**Cells**	**Dic**	**D_0_**	**D_1_**	**D_2_**	**D_3_**	**Yield**	**σ^2^/*y***	***u***
0	20,400	21	20,379	21	0	0	0.0010	1.00	−0.10
0.1	4,799	10	4,789	10	0	0	0.0021	1.00	−0.10
0.25	4,528	28	4,500	28	0	0	0.0062	0.99	−0.29
0.5	4,159	58	4,102	56	1	0	0.014	1.02	0.96
0.75	4,162	94	4,069	92	1	0	0.023	1.00	−0.05
1	4,540	162	4,380	158	2	0	0.036	0.99	−0.51
2	2,239	243	2,009	217	13	0	0.11	1.00	−0.04
3	1,059	241	846	185	28	0	0.23	1.01	0.13
4	1,054	349	765	234	50	5	0.33	1.04	0.97

Dic, the number of dicentrics; D_*n*_, number of cells with *n* dicentric(s).

### Dose estimates for *ex vivo* irradiated blind samples

**Figure 2:**
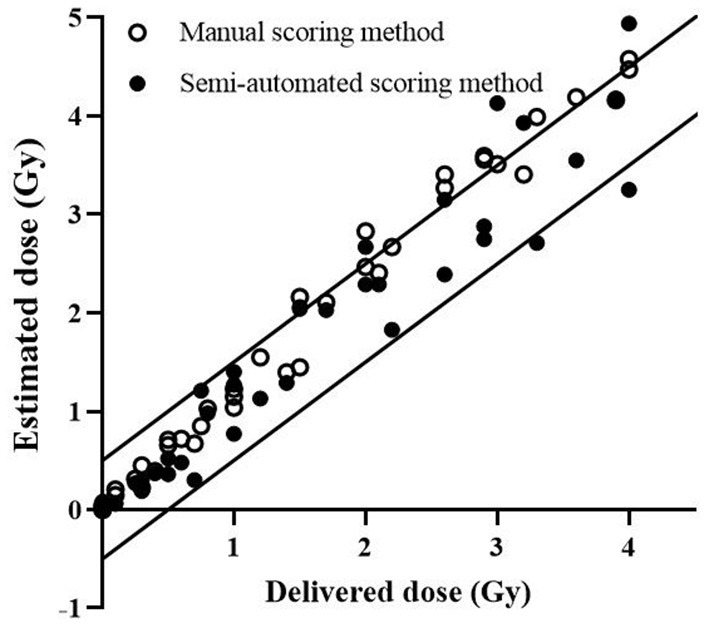
Estimated doses by DCScore software. Each data point represents the dose estimate by conventional manual DCA (open circles) and DCScore-based semi-automated scoring method (filled circles). The solid lines represent ±0.5 Gy intervals.

Dose estimations with dicentric yields of the blind samples were performed using the semi-automated scoring method, and the dose estimation accuracy was compared with the manual DCA results (Supplementary Table 1). The delivered dose of most blind samples [37 of 39 samples (94.9%)] fell within the 95% CI of the dose estimates, using the semi-automated scoring method. The number of dose estimates lying outside the 0.5-Gy uncertainty interval, accepted for triage dosimetry based on DCA, were similar for the manual and semi-automated scoring methods [10 of 39 samples (25.6%) for manual DCA; 9 of 39 samples (23.1%) for the semi-automated scoring method; Figure 2]. In addition, there was no difference in mean absolute deviation (MAD) values between the two methods (0.29 for manual DCA vs. 0.28 for the semi-automated scoring method).

We tested the performance of the automatic scoring system to classify persons depending on their need for acute clinical intervention, more detailed diagnostic tests, or long-term epidemiological follow-up (26). Table 2 shows the performance of the automatic scoring system within such a framework. The semi-automated scoring method with the DCScore module also had more than 90% accuracy, sensitivity, and specificity to distinguish binary dose categories reflecting clinical, diagnostic, and epidemiological significance. Thus, the assay performance of the DCScore-based scoring system was comparable to that of manual DCA.

**Table 2:** Accuracy, sensitivity, and specificity of triage classification.

**Radiation exposure**	**Accuracy**	**Sensitivity**	**Specificity**
**Manual scoring method**			
Never vs. Ever	100	100	100
≤ 0.1 vs. > 0.1 Gy	95	100	66.7
≤ 1.5 vs. > 1.5 Gy	95	100	91.3
**Semi-automated scoring method**			
Never vs. Ever	95	94.3	100
≤ 0.1 vs. > 0.1 Gy	100	100	100
≤ 1.5 vs. > 1.5 Gy	95	100	91.3

### Comparison of the manual and semi-automated scoring methods in occupationally exposed persons

**Figure 3:**
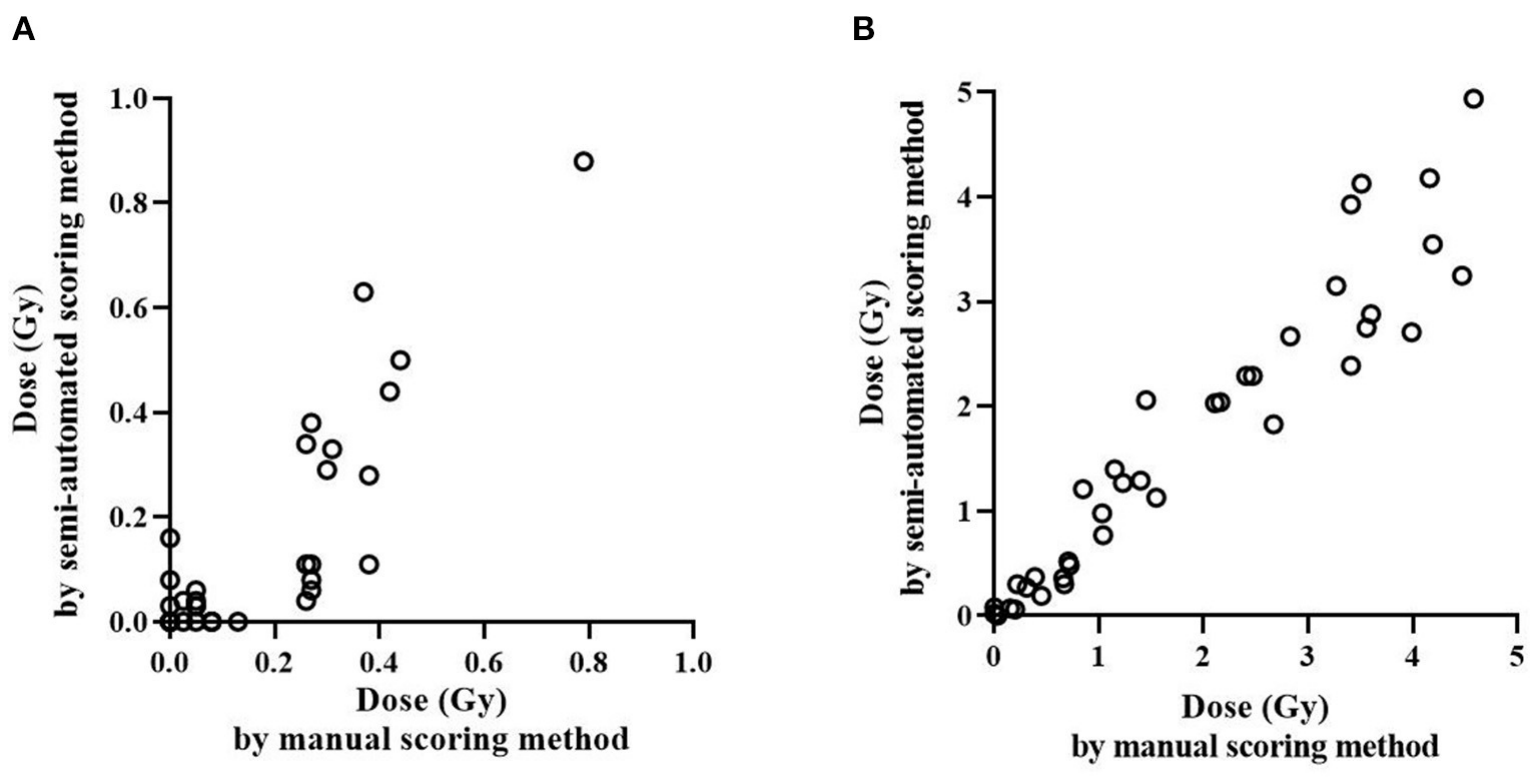
Comparison of radiation dose estimation by the manual and semi-automated scoring methods. Irradiated dose of *ex vivo* and *in vivo* samples were estimated and compared using the manual and semi-automated scoring method. Dose estimation of *in vivo* occupationally exposed samples **(A)** and *ex vivo* irradiated samples **(B)** is shown.

To investigate whether the semi-automated scoring method can be useful for *in vivo* irradiated samples, the dose estimates of occupationally exposed persons using manual and semi-automated scoring methods were compared (Supplementary Table 2). First, dose estimates by each method were categorized into binary categories using a threshold dose of 0.1 Gy, which can distinguish never vs. ever radiation exposure (≥0.1 Gy) as shown in Table 3. The kappa value was 0.71 (*p* < 0.0001) and McNemar's test was not significant (*p* = 0.18), which indicates that there was a substantial agreement between the results of the two methods without systematic difference. Figure 3 shows how the dose estimates using manual and the semi-automated scoring method agree. There was a significant correlation between the two methods in *in vivo* occupationally exposed samples (correlation coefficient = 0.85, *p* < 0.0001). Samples irradiated *ex vivo* also showed significant correlation (correlation coefficient = 0.97, *p* < 0.0001).

**Table 3:** Contingency table showing the agreement between manual and semi-automated scoring method in occupationally exposed persons.

	**Manual scoring method**		
**Semi-automated** **scoring method**	** < 0.1 Gy**,	**≥0.1 Gy**,	**Total**,
	***N* (%)**	***N* (%)**	***N* (%)**
< 0.1 Gy	18 (81.8)	4 (18.2)	22 (100.0)
≥0.1Gy	1 (7.7)	12 (92.3)	13 (100.0)
Total	19 (54.3)	16 (45.7)	35 (100.0)

Kappa = 0.71 (*p* < 0.0001); McNemar's test *p* = 0.18.

## Discussion

In this study, we compared the dose estimations of DCScore and manual DCA using *ex vivo* and *in vivo* irradiated samples. Although it is known that automated scoring using DCScore causes scoring error compared to manual DCA, it could be acceptable to use if the method can provide reliable dose estimation from its calibration curve. Furthermore, we adapted the semi-automated scoring method for DCScore to reduce scoring error of automated method and improve the accuracy as much as possible, and tested the ability to estimate dose. We confirmed that the semi-automated scoring method can provide rapid and accurate results, and thus, serve as an alternative to manual DCA for radiation biodosimetry.

According to Schunk et al. (11), DCScore overestimates the number of DC, especially at doses below 2 Gy. Moreover, attached or overlapping chromosomes can be detected as dicentrics in DCScore. To reduce false positives caused by DCScore, most researchers have chosen semi-automated scoring methods to validate dicentrics detected by DCScore (9, 22). We also inspected the DC after using DCScore, and these chromosomes, confirmed by well-trained scorers, were used to construct the dose-response curve and estimate the doses. Although additional time is required to validate dicentrics, only a few minutes would be taken, given that DC frequency is generally low. Whereas, it generally takes between 6 and 8 h for analyzing 500 metaphase cells of one sample in manual DCA scoring (27), validating around 200 dicentrics for one sample took <10 min in this study. Romm et al. (9) also analyzed dicentrics in approximately 20 min per sample, which was much shorter than the time for triage mode (50 cells in 60 min by one scorer). Therefore, the semi-automated scoring method is a rapid and efficient alternative to manual DCA in estimating doses.

The distribution of dicentrics in irradiated cells has been known to follow a Poisson distribution (1). Dose estimation and the uncertainty of testing samples are calculated assuming that the dicentrics in the samples follow a Poisson distribution. Therefore, when performing dose estimation, assessing the conformity of the distribution of dicentrics to the Poisson distribution is important. In our study, all samples used in constructing the dose response curve had a *U*-value (a normalized unit of dispersion index) of <1.96. This finding, which indicates that the distribution of dicentrics detected by the semi-automated scoring method also follows a Poisson distribution, is consistent with the findings by Romm et al. (9) and Vaurijoux et al. (28). Similar to previous studies, DC frequencies in the semi-automated scoring method were lower than those in the manual scoring method. This result can be explained by the higher probability of missing a dicentric in the automated scoring method than in the manual scoring method (9).

In this study, we compared the dose estimates of 39 blind samples tested in intercomparison exercises using the two scoring methods. The semi-automated scoring method has high sensitivity, specificity, and accuracy for discriminating binary categories of clinical significance. The discrimination ability of the semi-automated scoring method was comparable to that of our manual DCA and those of the scoring methods in other cytogenetic labs (29). Indeed, the semi-automated scoring method estimated accurate doses of most samples (94.9% accuracy), suggesting that our semi-automated scoring method could identify exposed individuals who will probably suffer from acute radiation syndrome several days after radiation exposure or will have deterministic or stochastic effects.

The strength of this study lies in the evaluation of the performance of the semi-automated scoring method using occupationally exposed samples *in vivo*. Radiation workers wear legal personal dosimeters during work to monitor their radiation exposure, but performing biodosimetry is necessary due to low personal dosimeter-wearing compliance and loss or damage of dosimeters. Since 2010, our laboratory has performed DCA for estimating the radiation dose in hundreds of radiation workers with errors in physical dosimetry or suspicions of over-exposure. Most of the radiation doses are generally lower than those assessed in *in vitro* testing or radiological accidents. To investigate the application of the semi-automated scoring method in monitoring occupationally exposed individuals, dose estimates of radiation workers using the manual and the semi-automated scoring methods were compared. Our results showed a strong agreement between the abilities of the two methods to distinguish workers exposed to <0.1 and ≥0.1 Gy, suggesting that our semi-automated scoring method is a suitable alternative to manual DCA in identifying exposed individuals from the “worried well” group. Indeed, analysis of *in vivo* irradiated samples showed a correlation between the two methods with statistical significance. However, we need to consider our *in vivo* radiation workers were exposed to relatively higher radiation dose. In order to assess the ability to estimate dose in wide range of radiation dose, workers exposed to high and low radiation exposure were randomly selected and their metaphase images were reanalyzed using semi-automated scoring method. Due to the reason, the dose range of *in vivo* cohort we tested could be relatively higher than actual occupational radiation exposure. In addition, we took dose estimates of samples with 0 dicentrics or negative dose estimates as 0, there might be possible to overestimate the statistical values. Therefore, the performance of semi-automated scoring should be assessed in low dose radiation exposed population in further studies.

We didn't compare the physical dose and biological dose estimated by DCScore for our *in vivo* cohort. The objective of this study was to investigate whether semi-automated method is comparable to manual scoring method. Due to the reason, we compared results of semi-automated and manual scoring methods only without physical dosimetry information here, by analyzing same image pools for both methods. Further studies to compare their physical dose and biological dose could be used to assess personal dosimeter-wearing compliance of radiation workers as well as dose estimation with automated methods.

A limitation of scoring systems with DCScore is that metaphase images that are poor quality are rejected and few images that are good quality are accepted for scoring. The quality of metaphase spreads is an important factor that affects DC scoring using DCScore (9, 21). As our study utilized images generated in previous studies for validating the semi-automated scoring method, we could not adjust the image quality and used a smaller number of metaphase images in the semi-automated scoring method than in the manual scoring method. Although the DC frequency was scored in around 500 cells of 1,000 image pools (50% rejection rate) due to the aforementioned reasons, the rejection rate is similar to those of methods used in other laboratories (9). Indeed, the difference between the estimated and true doses was similar between the manual and semi-automated scoring methods. Further studies to improve image quality would help increase the number of scorable metaphase images in DCScore. Another limitation is the lower sensitivity of semi-automated scoring method. Efforts to increase the number of metaphase cells for analysis could improve its sensitivity (30). Dose estimation of more samples exposed to low dose radiation could confirm the capability of semi-automated scoring methods to distinguish low-dose range.

Our study found that the performance of the semi-automated scoring method in estimating the radiation dose is comparable to that of manual DCA. Although the semi-automated scoring method requires an additional examination to correct false positive results, this method could be sufficient to reduce scoring time and increase throughput compared to manual DCA. Whereas, manual DCA takes one working day for analyzing 1,000 metaphases by two scorers, the semi-automated scoring method can re-examine the DCScore results within a few minutes, as it only checks for the dicentric images. Therefore, the semi-automated scoring method, which can provide accurate dose estimations rapidly, can be useful as an alternative to manual DCA for biodosimetry in large-scale accidents or cases to monitor radiation exposure of radiation workers. Despite of comparable results with two methods, lower sensitivity of semi-automated scoring method and higher radiation exposure in our *in vivo* cohorts could be limited to assess various radiation exposures. Taken together, our findings show the semi-automated scoring method can provide accurate dose estimation rapidly, and can be useful as an alternative to manual DCA for biodosimetry in large-scale accidents. Further efforts to increase the number of metaphase cells for analysis and assess dose estimation of low dose exposed cohorts could confirm our findings and thus semi-automated scoring method could be applied to monitor various radiation conditions including low-dose exposure.

This study used a semi-automated scoring method because of the high false positive rate of automated scoring systems. Further efforts to reduce the errors of automated scoring systems could improve the throughput and accuracy of automated scoring, which makes use of artificial intelligence machine learning.

## Data availability statement

The original contributions presented in the study are included in the article/Supplementary material, further inquiries can be directed to the corresponding author/s.

## Ethics statement

The studies involving human participants were reviewed and approved by Korea Institute of Radiological and Medical Sciences, Institutional Review Board and the Health Canada and Public Health Agency of Canada's Research Ethics Board. The patients/participants provided their written informed consent to participate in this study.

## Author contributions

SJ contributed to conception and design of the study, analyzed the data, and edited the manuscript. YL analyzed the data, made figures, and wrote the manuscript. SK, YHL, SY, and HY contributed to the data collection. RW contributed to the data collection and manuscript edits. All authors contributed to manuscript revision, read, and approved the submitted version.

## Funding

This work was supported by the Nuclear Safety and Security Commission Republic of Korea (grant no. 1803014) and a grant from the Korea Institute of Radiological and Medical Sciences (KIRAMS), funded by the Ministry of Science and ICT (MSIT), and Republic of Korea (No. 50445-2022).

## Conflict of interest

The authors declare that the research was conducted in the absence of any commercial or financial relationships that could be construed as a potential conflict of interest.

## Publisher's note

All claims expressed in this article are solely those of the authors and do not necessarily represent those of their affiliated organizations, or those of the publisher, the editors and the reviewers. Any product that may be evaluated in this article, or claim that may be made by its manufacturer, is not guaranteed or endorsed by the publisher.
