# Supplementary material for: Application of a semi-automated dicentric scoring system in triage and monitoring occupational radiation exposure
Source: Front Public Health. 2022 Oct 20;10:1002501. doi: 10.3389/fpubh.2022.1002501 (PMC9631783; doi:10.3389/fpubh.2022.1002501)
Supplement: Supplementary file 1 [file Data_Sheet_1.docx]

Supplementary Material

Supplementary Table 1. Dose estimation results of *in vitro* irradiated blind samples according to scoring methods

| Sample ID | metaphases (n) | Dicentrics (n) | DC frequency (per metaphase) | Delivered dose (Gy) | Dose estimation (Gy) | | |
| --- | --- | --- | --- | --- | --- | --- | --- |
|  |  |  |  |  | Dose | 95% LL | 95%UL |
| Semi-automated scoring method | | | | |  |  |  |
| ILC#1 | 310 | 31 | 0.100 | 1.50 | 2.04 | 1.02 | 3.48 |
| ILC#2 | 802 | 1 | 0.001 | 0.00 | 0.02 | 0.00 | 0.30 |
| ILC#3 | 248 | 100 | 0.403 | 4.00 | 4.94 | 3.53 | 6.85 |
| ILC#4 | 590 | 5 | 0.008 | 0.25 | 0.27 | 0.03 | 0.72 |
| ILC#5 | 912 | 44 | 0.048 | 0.75 | 1.21 | 0.80 | 1.68 |
| ILC#6 | 475 | 8 | 0.017 | 0.50 | 0.52 | 0.15 | 1.04 |
| ILC#7 | 237 | 71 | 0.300 | 3.00 | 4.13 | 2.81 | 6.01 |
| ILC#8 | 788 | 2 | 0.003 | 0.10 | 0.07 | 0.00 | 0.38 |
| ILC#9 | 205 | 12 | 0.059 | 1.00 | 1.40 | 0.43 | 3.16 |
| ILC#10 | 186 | 22 | 0.118 | 2.00 | 2.29 | 1.49 | 3.16 |
| ILC#11 | 175 | 2 | 0.011 | 0.50 | 0.36 | 0.00 | 1.19 |
| ILC#12 | 331 | 101 | 0.305 | 3.90 | 4.18 | 2.99 | 5.74 |
| ILC#13 | 686 | 35 | 0.051 | 1.00 | 1.27 | 0.80 | 1.76 |
| ILC#14 | 706 | 0 | 0.000 | 0.00 | -0.03 |  |  |
| ILC#15 | 198 | 20 | 0.101 | 1.50 | 2.06 | 1.34 | 2.90 |
| ILC#16 | 428 | 4 | 0.009 | 0.30 | 0.30 | 0.02 | 0.82 |
| ILC#17 | 526 | 5 | 0.010 | 0.70 | 0.30 | 0.04 | 0.78 |
| ILC#18 | 273 | 46 | 0.168 | 2.90 | 2.88 | 2.20 | 3.69 |
| ILC#19 | 802 | 2 | 0.002 | 0.10 | 0.06 | 0.00 | 0.37 |
| ILC#20 | 668 | 79 | 0.118 | 2.10 | 2.29 | 1.49 | 3.26 |
| ILC#21 | 618 | 27 | 0.044 | 1.20 | 1.13 | 0.67 | 1.63 |
| ILC#22 | 217 | 34 | 0.157 | 2.90 | 2.75 | 2.00 | 3.65 |
| ILC#23 | 371 | 0 | 0.000 | 0.00 | 0.00 |  |  |
| ILC#24 | 507 | 64 | 0.126 | 2.60 | 2.39 | 1.48 | 3.45 |
| ILC#25 | 645 | 10 | 0.016 | 0.60 | 0.48 | 0.19 | 0.94 |
| ILC#26 | 433 | 43 | 0.099 | 1.70 | 2.03 | 1.15 | 3.18 |
| ILC#27 | 229 | 35 | 0.153 | 3.30 | 2.71 | 1.93 | 3.55 |
| ILC#28 | 360 | 73 | 0.203 | 4.00 | 3.25 | 2.59 | 4.01 |
| ILC#29 | 502 | 3 | 0.006 | 0.30 | 0.19 | 0.00 | 0.64 |
| ILC#30 | 1458 | 76 | 0.052 | 1.40 | 1.29 | 0.91 | 1.68 |
| ILC#31 | 362 | 100 | 0.276 | 3.20 | 3.93 | 2.80 | 5.39 |
| ILC#32 | 1104 | 13 | 0.012 | 0.40 | 0.37 | 0.13 | 0.73 |
| ILC#33 | 516 | 100 | 0.194 | 2.60 | 3.15 | 2.19 | 4.32 |
| ILC#34 | 674 | 100 | 0.148 | 2.00 | 2.67 | 2.14 | 3.23 |
| ILC#35 | 2408 | 7 | 0.003 | 0.00 | 0.08 | 0.00 | 0.27 |
| ILC#36 | 994 | 27 | 0.027 | 1.00 | 0.77 | 0.43 | 1.19 |
| ILC#37 | 428 | 100 | 0.234 | 3.60 | 3.55 | 2.50 | 4.86 |
| ILC#38 | 2757 | 100 | 0.036 | 0.80 | 0.98 | 0.68 | 1.30 |
| ILC#39 | 515 | 44 | 0.085 | 2.20 | 1.83 | 0.98 | 2.91 |
| Manual scoring method | | | | |  |  |  |
| ILC#1 | 253 | 100 | 0.40 | 1.50 | 2.16 | 1.63 | 2.76 |
| ILC#2 | 1000 | 1 | 0.00 | 0.00 | 0.00 | 0.00 | 0.25 |
| ILC#3 | 62 | 101 | 1.63 | 4.00 | 4.58 | 3.92 | 5.31 |
| ILC#4 | 1000 | 17 | 0.02 | 0.25 | 0.31 | 0.16 | 0.49 |
| ILC#5 | 1000 | 76 | 0.08 | 0.75 | 0.85 | 0.67 | 1.02 |
| ILC#6 | 1000 | 57 | 0.06 | 0.50 | 0.71 | 0.45 | 1.02 |
| ILC#7 | 102 | 100 | 0.98 | 3.00 | 3.51 | 3.00 | 4.07 |
| ILC#8 | 1000 | 7 | 0.01 | 0.10 | 0.15 | 0.00 | 0.52 |
| ILC#9 | 440 | 56 | 0.13 | 1.00 | 1.15 | 0.75 | 1.59 |
| ILC#10 | 212 | 102 | 0.48 | 2.00 | 2.47 | 2.13 | 2.84 |
| ILC#11 | 1000 | 52 | 0.05 | 0.50 | 0.66 | 0.38 | 0.96 |
| ILC#12 | 79 | 100 | 1.27 | 3.90 | 4.16 | 3.56 | 4.81 |
| ILC#13 | 706 | 100 | 0.14 | 1.00 | 1.23 | 1.03 | 1.42 |
| ILC#14 | 1000 | 3 | 0.00 | 0.00 | 0.05 | 0.00 | 0.19 |
| ILC#15 | 533 | 100 | 0.19 | 1.50 | 1.45 | 1.07 | 1.86 |
| ILC#16 | 1000 | 12 | 0.01 | 0.30 | 0.22 | 0.05 | 0.56 |
| ILC#17 | 1000 | 54 | 0.05 | 0.70 | 0.67 | 0.06 | 0.84 |
| ILC#18 | 105 | 101 | 0.96 | 2.90 | 3.60 | 3.09 | 4.14 |
| ILC#19 | 1000 | 11 | 0.01 | 0.10 | 0.20 | 0.08 | 0.36 |
| ILC#20 | 220 | 101 | 0.46 | 2.10 | 2.41 | 1.83 | 3.06 |
| ILC#21 | 475 | 100 | 0.21 | 1.20 | 1.55 | 1.14 | 2.00 |
| ILC#22 | 107 | 101 | 0.94 | 2.90 | 3.56 | 3.06 | 4.10 |
| ILC#23 | 1000 | 2 | 0.00 | 0.00 | 0.03 | 0.00 | 0.16 |
| ILC#24 | 115 | 100 | 0.87 | 2.60 | 3.41 | 2.58 | 4.36 |
| ILC#25 | 1000 | 60 | 0.06 | 0.60 | 0.72 | 0.45 | 1.02 |
| ILC#26 | 276 | 100 | 0.36 | 1.70 | 2.11 | 1.58 | 2.70 |
| ILC#27 | 89 | 104 | 1.17 | 3.30 | 3.99 | 3.46 | 4.63 |
| ILC#28 | 69 | 100 | 1.45 | 4.00 | 4.47 | 3.82 | 5.18 |
| ILC#29 | 1000 | 30 | 0.03 | 0.30 | 0.45 | 0.30 | 0.61 |
| ILC#30 | 567 | 100 | 0.18 | 1.40 | 1.40 | 1.18 | 1.62 |
| ILC#31 | 115 | 100 | 0.87 | 3.20 | 3.41 | 2.91 | 3.93 |
| ILC#32 | 1000 | 25 | 0.03 | 0.40 | 0.39 | 0.25 | 0.56 |
| ILC#33 | 124 | 100 | 0.81 | 2.60 | 3.27 | 2.80 | 3.78 |
| ILC#34 | 162 | 100 | 0.62 | 2.00 | 2.83 | 2.14 | 3.62 |
| ILC#35 | 1000 | 1 | 0.00 | 0.00 | 0.00 | 0.00 | 0.25 |
| ILC#36 | 930 | 100 | 0.11 | 1.00 | 1.04 | 0.74 | 1.36 |
| ILC#37 | 78 | 100 | 1.28 | 3.60 | 4.19 | 3.58 | 4.85 |
| ILC#38 | 947 | 100 | 0.11 | 0.80 | 1.03 | 0.73 | 1.34 |
| ILC#39 | 180 | 100 | 0.56 | 2.20 | 2.67 | 2.29 | 3.08 |
|  |  |  |  |  |  |  |  |

When actual delivered dose fell within 95% confidence interval of a dose estimate, we considered it is correctly estimated.Supplementary Table 2. Dose estimation results of occupationally exposed persons according to scoring methods

| Sample ID | Metaphases (n) | Dicentrics (n) | DC frequency (per metaphase) | Dose estimation (Gy) | | |
| --- | --- | --- | --- | --- | --- | --- |
|  |  |  |  | Dose | 95% LL | 95%UL |
| Semi-automated scoring method | | | | | | |
| W#1 | 534 | 17 | 0.032 | 0.88 | 0.44 | 1.41 |
| W#2 | 365 | 6 | 0.016 | 0.50 | 0.16 | 1.12 |
| W#3 | 428 | 6 | 0.014 | 0.44 | 0.00 | 1.80 |
| W#4 | 527 | 2 | 0.004 | 0.11 | 0.00 | 0.53 |
| W#5 | 347 | 3 | 0.009 | 0.28 | 0.00 | 0.84 |
| W#6 | 517 | 11 | 0.021 | 0.63 | 0.25 | 1.16 |
| W#7 | 391 | 4 | 0.010 | 0.33 | 0.03 | 0.87 |
| W#8 | 783 | 7 | 0.009 | 0.29 | 0.06 | 0.68 |
| W#9 | 341 | 1 | 0.003 | 0.08 | 0.00 | 0.62 |
| W#10 | 586 | 7 | 0.012 | 0.38 | 0.11 | 0.84 |
| W#11 | 264 | 1 | 0.004 | 0.11 | 0.00 | 0.75 |
| W#12 | 413 | 1 | 0.002 | 0.06 | 0.00 | 0.53 |
| W#13 | 531 | 1 | 0.002 | 0.04 | 0.00 | 0.43 |
| W#14 | 551 | 2 | 0.004 | 0.11 | 0.00 | 0.51 |
| W#15 | 469 | 5 | 0.011 | 0.34 | 0.06 | 0.85 |
| W#16 | 617 | 0 | 0.000 | 0.00 |  |  |
| W#17 | 417 | 1 | 0.002 | 0.06 | 0.00 | 0.53 |
| W#18 | 163 | 0 | 0.000 | 0.00 |  |  |
| W#19 | 579 | 3 | 0.005 | 0.16 | 0.00 | 0.57 |
| W#20 | 111 | 0 | 0.000 | 0.00 |  |  |
| W#21 | 629 | 1 | 0.002 | 0.03 | 0.00 | 0.37 |
| W#22 | 474 | 0 | 0.000 | 0.00 |  |  |
| W#23 | 346 | 0 | 0.000 | 0.00 |  |  |
| W#24 | 680 | 1 | 0.001 | 0.03 | 0.00 | 0.35 |
| W#25 | 432 | 0 | 0.000 | 0.00 |  |  |
| W#26 | 431 | 0 | 0.000 | 0.00 |  |  |
| W#27 | 601 | 1 | 0.002 | 0.03 | 0.00 | 0.39 |
| W#28 | 553 | 1 | 0.002 | 0.04 | 0.00 | 0.42 |
| W#29 | 352 | 0 | 0.000 | 0.00 |  |  |
| W#30 | 425 | 0 | 0.000 | 0.00 |  |  |
| W#31 | 561 | 1 | 0.002 | 0.04 | 0.00 | 0.41 |
| W#32 | 627 | 0 | 0.000 | 0.00 |  |  |
| W#33 | 593 | 0 | 0.000 | 0.00 |  |  |
| W#34 | 502 | 0 | 0.000 | 0.00 |  |  |
| W#35 | 346 | 1 | 0.003 | 0.08 | 0.00 | 0.61 |
|  | | | | | | |
|  | | | | | | |
| Manual scoring method | | | | | | |
| W#1 | 1000 | 68 | 0.068 | 0.79 | 0.62 | 0.96 |
| W#2 | 1000 | 27 | 0.027 | 0.44 | 0.17 | 0.78 |
| W#3 | 1000 | 25 | 0.025 | 0.42 | 0.26 | 0.59 |
| W#4 | 1000 | 22 | 0.022 | 0.38 | 0.14 | 0.70 |
| W#5 | 1000 | 22 | 0.022 | 0.38 | 0.22 | 0.55 |
| W#6 | 1000 | 21 | 0.021 | 0.37 | 0.21 | 0.55 |
| W#7 | 1000 | 17 | 0.017 | 0.31 | 0.16 | 0.49 |
| W#8 | 1000 | 16 | 0.016 | 0.30 | 0.16 | 0.48 |
| W#9 | 1000 | 14 | 0.014 | 0.27 | 0.05 | 0.65 |
| W#10 | 1000 | 14 | 0.014 | 0.27 | 0.06 | 0.62 |
| W#11 | 1000 | 14 | 0.014 | 0.27 | 0.13 | 0.45 |
| W#12 | 1000 | 14 | 0.014 | 0.27 | 0.05 | 0.64 |
| W#13 | 1000 | 13 | 0.013 | 0.26 | 0.11 | 0.43 |
| W#14 | 1000 | 13 | 0.013 | 0.26 | 0.11 | 0.43 |
| W#15 | 1000 | 13 | 0.013 | 0.26 | 0.11 | 0.43 |
| W#16 | 1000 | 3 | 0.003 | 0.05 | 0.00 | 0.19 |
| W#17 | 1000 | 3 | 0.003 | 0.05 | 0.00 | 0.19 |
| W#18 | 1000 | 4 | 0.004 | 0.08 | 0.00 | 0.25 |
| W#19 | 1000 | 0 | 0.000 | 0.00 |  |  |
| W#20 | 1000 | 4 | 0.004 | 0.08 | 0.00 | 0.25 |
| W#21 | 1000 | 3 | 0.003 | 0.05 | 0.00 | 0.19 |
| W#22 | 1000 | 4 | 0.004 | 0.08 | 0.00 | 0.25 |
| W#23 | 1000 | 6 | 0.006 | 0.13 | 0.00 | 0.31 |
| W#24 | 1000 | 3 | 0.003 | 0.05 | 0.00 | 0.19 |
| W#25 | 1000 | 1 | 0.001 | 0.00 | 0.00 | 0.13 |
| W#26 | 1000 | 2 | 0.002 | 0.026 | 0.00 | 0.16 |
| W#27 | 1000 | 1 | 0.001 | 0.00 | 0.00 | 0.13 |
| W#28 | 1000 | 3 | 0.003 | 0.05 | 0.00 | 0.19 |
| W#29 | 1000 | 1 | 0.001 | 0.00 | 0.00 | 0.13 |
| W#30 | 1000 | 1 | 0.001 | 0.00 | 0.00 | 0.13 |
| W#31 | 1000 | 2 | 0.002 | 0.026 | 0.00 | 0.16 |
| W#32 | 1000 | 1 | 0.001 | 0.00 | 0.00 | 0.13 |
| W#33 | 1000 | 0 | 0.000 | 0.00 |  |  |
| W#34 | 1000 | 1 | 0.001 | 0.00 | 0.00 | 0.13 |
| W#35 | 1000 | 0 | 0.000 | 0.00 |  |  |
|  |  |  |  |  |  |  |

Dose estimates of 0 dicentrics and negative values were taken as 0.
